# Crystal structure of 4-(4-chloro­phen­yl)-6-(morpholin-4-yl)pyridazin-3(2*H*)-one

**DOI:** 10.1107/S2056989015012980

**Published:** 2015-07-17

**Authors:** Abdullah Aydın, Mehmet Akkurt, Murat Şüküroğlu, Orhan Büyükgüngör

**Affiliations:** a Department of Science Education, Faculty of Education, Kastamonu University, 37200 Kastamonu, Turkey; b Department of Physics, Faculty of Sciences, Erciyes University, 38039 Kayseri, Turkey; c Department of Pharmaceutical Chemistry, Faculty of Pharmacy, Gazi University, 06330 Ankara, Turkey; d Department of Physics, Faculty of Arts and Sciences, Ondokuz Mayıs University, 55139 Samsun, Turkey

**Keywords:** crystal structure, pyridazinone derivative, hydrogen bonding, π–π stacking inter­actions

## Abstract

In the title compound, C_14_H_14_ClN_3_O_2_, the morpholine ring adopts a chair conformation, with the exocyclic N—C bond in an equatorial orientation. The 1,6-di­hydro­pyridazine ring is essentially planar, with a maximum deviation of 0.014 (1) Å, and forms a dihedral angle of 40.16 (7)° with the plane of the benzene ring. In the crystal, pairs of centrosymmetrically related mol­ecules are linked into dimers *via* N—H⋯O hydrogen bonds, forming *R*_2_^2^(8) ring motifs. The dimers are connected *via* C—H⋯O and C—H⋯Cl hydrogen bonds, forming a three-dimensional network. Aromatic π–π stacking inter­actions [centroid–centroid distance = 3.6665 (9) Å] are also observed. Semi-empirical mol­ecular orbital calculations were carried out using the AM1 method. The calculated dihedral angles between the pyridizine and benzene rings and between the pyridizine and morpholine (all atoms) rings are 34.49 and 76.96°, respectively·The corresponding values obtained from the X-ray structure determination are 40.16 (7) and 12.97 (9)°, respectively. The morpholine ring of the title compound in the calculated gas-phase seems to have a quite different orientation compared to that indicated by the X-ray structure determination.

## Abstract 2

In the crystal, pairs of centrosymmetrically related mol­ecules are linked into dimers *via* N—H⋯O hydrogen bonds, forming 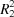
(8) ring motifs. The dimers are connected *via* C—H⋯O and C—H⋯Cl hydrogen bonds, forming a three-dimensional network·Semi-empirical mol­ecular orbital calculations were carried out using the AM1 method.

## Chemical context   

The title compound was first synthesized by Şüküroğlu *et al.* (2006 ▸). The pharmacological properties of the compound have been investigated and it was found it possesses an analgesic effect close to that of aspirin. In recent years, the 3(2*H*)-pyridazinone system has aroused a great deal of attention due to its structural relationship to pyrazolone derivatives such as amino­pyrine and dipyrone in view of the ring enlargement of pyrazolone to pyridazinone. These drugs possess analgesic and anti-inflammatory activities although they have limitations for their clinical use due to serious side effects such as blood dyscrasias (Şüküroğlu *et al.*, 2006 ▸; Brogden, 1986 ▸).
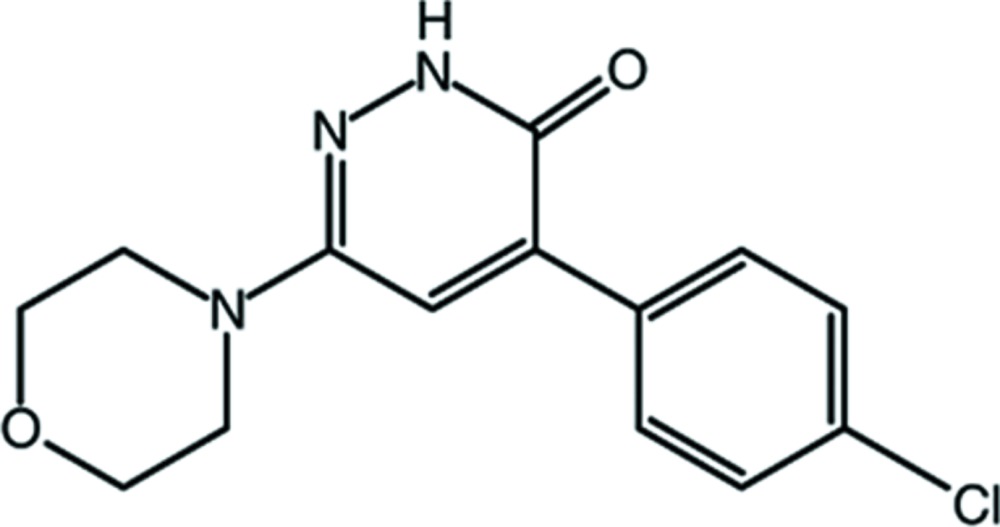


## Structural commentary   

**Figure 1:**
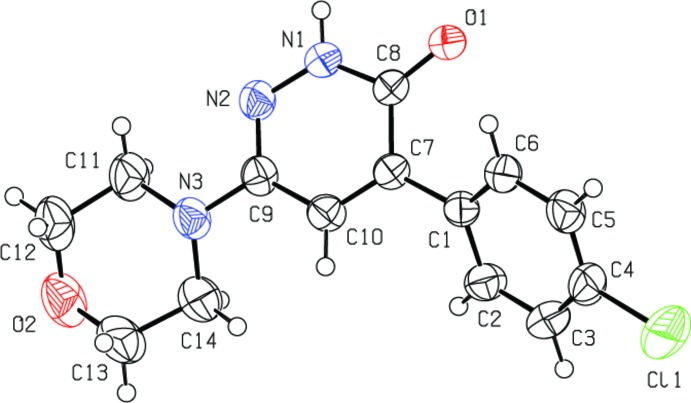
View of the title mol­ecule with displacement ellipsoids for non-H atoms drawn at the 30% probability level.

In the title compound (Fig. 1 ▸) the morpholine ring (N3/O2/C11–C14) adopts a chair conformation, with the puckering parameters *Q*_T_ = 0.551 (2) Å, θ = 174.33 (19) and ϕ = 175 (2)°. The 1,6-di­hydro­pyridazine ring (N1/N2/C7–C10) is essentially planar, with a maximum deviation of 0.014 (1) Å for atom N1 and forms a dihedral angle of 40.16 (7)° with the C1–C6 benzene ring. The dihedral angle between the morpholine ring (all atoms) and the pyridizine ring is 12.97 (9)°. The bond lengths and angles are in the normal range. The Cl1—C4, N1—N2 and O1—C12 bond lengths [1.7379 (17), 1.3620 (16), and 1.417 (3) Å, respectively] are consistent with those reported previously [1.753 (5), 1.275 (7) and 1.432 (7) Å in mol­ecule *A*; Aydin *et al.*, 2015 ▸]. In addition, the C8—O1 bond length of 1.2500 (16) Å compares well with the value of 1.2343 (17) Å reported by Aydın *et al.* (2011 ▸).

## Supra­molecular features   

**Figure 2:**
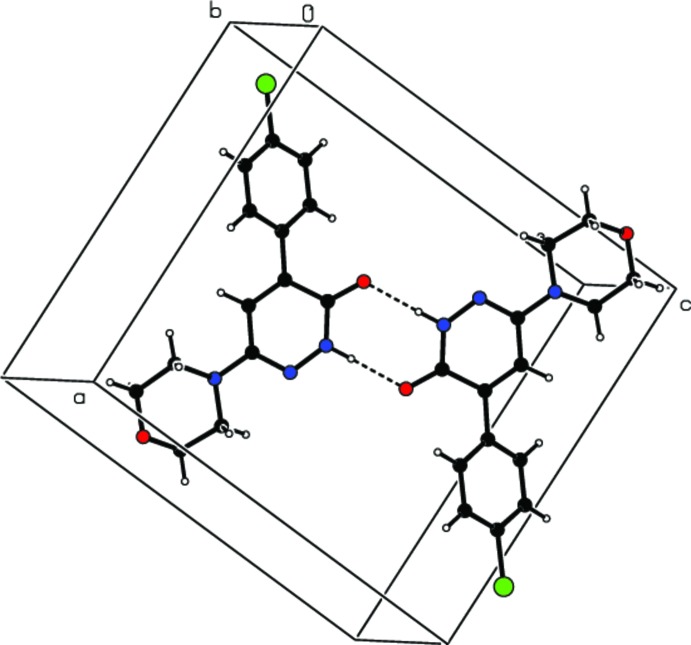
View of a dimer, with 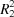
(8) ring motif, formed by N—H⋯O hydrogen bonds (dashed lines) between two centrosymmetrically related mol­ecules.

**Figure 3:**
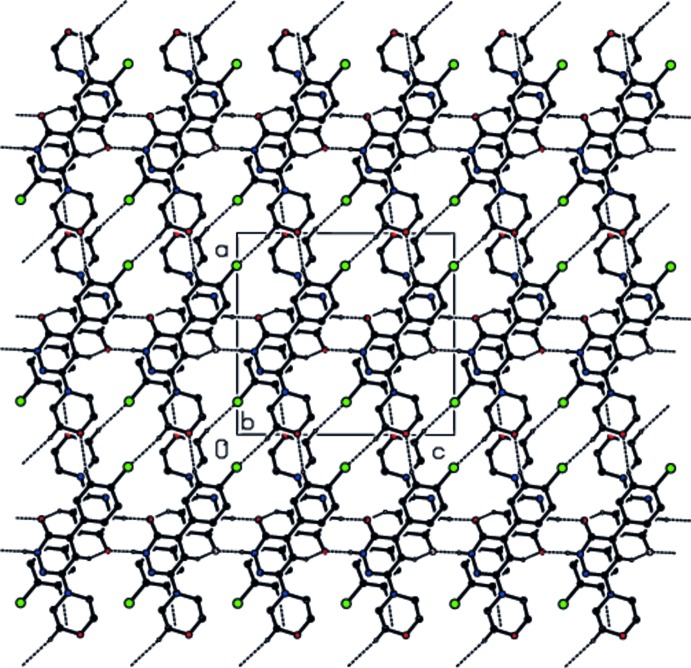
Crystal packing and hydrogen bonding (dashed lines) in the title compound, viewed down the *b* axis. H atoms not involved in hydrogen bonding have been omitted.

In the crystal, N—H⋯O hydrogen bonds (Table 1 ▸, Fig. 2 ▸) form dimers between centrosymmetric pairs of mol­ecules with 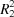
(8) ring motifs. The dimers are connected by C—H⋯O and C—H⋯Cl hydrogen bonds, forming a three-dimensional network (Table 1 ▸, Fig. 3 ▸). In addition, π–π stacking inter­actions [*Cg*2⋯*Cg*3^i^ = 3.6665 (9) Å; *Cg*2 and *Cg*3 are the centroids of the 1,6-di­hydro­pyridazine ring (N1/N2/C7–C10) and the benzene ring (C1–C6); symmetry code: (i) 1 − *x*, −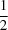 + *y*, 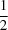 − *z*)] contribute to the cohesion of the structure.

## Semi-empirical mol­ecular orbital calculations   

**Figure 4:**
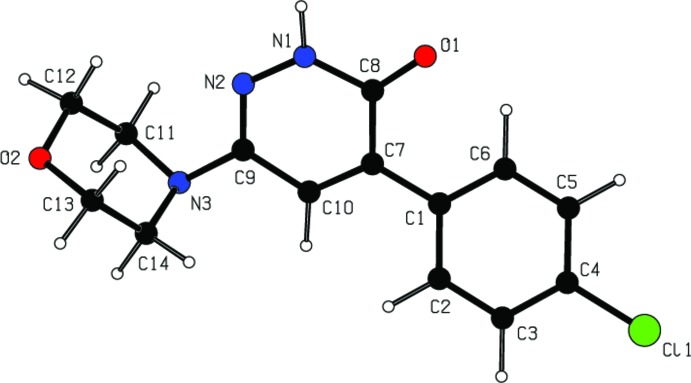
Spatial view of the title compound calculated using the AM1 method.

Semi-empirical mol­ecular orbital calculations of the title compound were carried out using the AM1 method (Dewar *et al.*, 1985 ▸) with *WinMopac7.2* software (Shchepin & Litvinov, 1998 ▸). A spatial view of the single mol­ecule of the title compound calculated in the gas phase is shown in Fig. 4 ▸. The calculated dihedral angles between the pyridizine and benzene rings and between the pyridizine and morpholine (all atoms) ring sare 34.49 and 76.96°, respectively. The corresponding values obtained from the X-ray structure determination are 40.16 (7) and 12.97 (9)°, respectively. The morpholine ring of the title compound in the calculated gas phase seems to have a quite different orientation compared to that indicated by the X-ray structure determination. The calculated dipole moment is 2.13 Debye. The HOMO and LUMO energy levels are −9.05 and −1.01 eV, respectively.

## Synthesis and crystallization   

4-(4-Chloro­phen­yl)-6-(morpholin-4-yl)pyridazin-3(2*H*)-one was prepared by a reported literature protocol (Şüküroğlu *et al.*, 2006 ▸). A solution of 3-chloro-4-phenyl-6-(morpholin-4-yl)-pyridazine (0.06 mol) and potassium acetate (0.08 mol) in 100 ml of acetic acid was refluxed for 10 h. The reaction mixture was then cooled and poured into ice–water. The precipitate was filtered off, washed with water and recrystallized from ethanol, giving yellow prismatic crystals. Yield 96%, m. p. 558 K. ^1^H NMR (CDCl_3_) δ 9.92 (*s*, 1H, NH), 7.76 (*m*, 2H, phenyl-H3, H5), 7.44 (*m*, 2H, phenyl-H2, H6), 7.23 (*s*, 1H, pyridazinone-H5), 3.85 (*t*, 4H, morpholine-H2, H6), 3.29 (*t*, 4H, morpholine-H3, H5) p.p.m. IR *v*_max_ cm^−1^ (KBr): 3124, 3053, 2960, 2861, 1656. Analysis C, H, N (C_14_H_14_ClN_3_O_2_)

## Refinement   

Crystal data, data collection and structure refinement details are summarized in Table 2 ▸. H atoms were positioned geometrically and refined using a riding model with N—H = 0.86 Å, C—H = 0.93–0.97 Å, and with *U*_iso_(H) = 1.2*U*_eq_(C, N).

## Supplementary Material

Crystal structure: contains datablock(s) global, I. DOI: 10.1107/S2056989015012980/rz5163sup1.cif

Structure factors: contains datablock(s) I. DOI: 10.1107/S2056989015012980/rz5163Isup2.hkl

Supporting information file. DOI: 10.1107/S2056989015012980/rz5163Isup3.cml

CCDC reference: 1410873

Additional supporting information:  crystallographic information; 3D view; checkCIF report

## Figures and Tables

**Table 1:** Hydrogen-bond geometry (, )

*D*H*A*	*D*H	H*A*	*D* *A*	*D*H*A*
N1H1O1^i^	0.86	1.92	2.7705(15)	170
C5H5O2^ii^	0.93	2.56	3.4737(19)	166
C10H10O1^iii^	0.93	2.43	3.1614(17)	136
C12H12*B*Cl1^iv^	0.97	2.79	3.754(2)	173

Symmetry codes: (i) 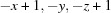; (ii) 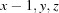; (iii) 
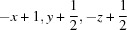
; (iv) 
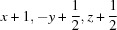
.

**Table 2:** Experimental details

Crystal data	
Chemical formula	C_14_H_14_ClN_3_O_2_
*M*_r_	291.73
Crystal system, space group	Monoclinic, *P*2_1_/*c*
Temperature (K)	296
*a*, *b*, *c* ()	13.0977(9), 7.4932(4), 14.1123(9)
()	90.149(5)
*V* (^3^)	1385.03(15)
*Z*	4
Radiation type	Mo *K*
(mm^1^)	0.28
Crystal size (mm)	0.80 0.38 0.08

Data collection	
Diffractometer	Stoe IPDS 2
Absorption correction	Integration (*X-RED32*; Stoe Cie, 2002 ▸)
*T*_min_, *T*_max_	0.880, 0.978
No. of measured, independent and observed [*I* > 2(*I*)] reflections	8760, 2868, 2267
*R*_int_	0.028
(sin /)_max_ (^1^)	0.628

Refinement	
*R*[*F* ^2^ > 2(*F* ^2^)], *wR*(*F* ^2^), *S*	0.039, 0.106, 1.04
No. of reflections	2868
No. of parameters	181
H-atom treatment	H-atom parameters constrained
_max_, _min_ (e ^3^)	0.14, 0.23

Computer programs: *X-AREA* and *X-RED32* (Stoe Cie, 2002 ▸), *SHELXS97* and *SHELXL97* (Sheldrick, 2008 ▸), *ORTEP-3 for Windows* and *WinGX* (Farrugia, 2012 ▸) and *PLATON* (Spek, 2009 ▸).

## supplementary crystallographic information

### Crystal data

**Table:**

C_14_H_14_ClN_3_O_2_	*F*(000) = 608
*M**_r_* = 291.73	*D*_x_ = 1.399 Mg m^−^^3^
Monoclinic, *P*2_1_/*c*	Mo *K*α radiation, λ = 0.71073 Å
Hall symbol: -P 2ybc	Cell parameters from 11553 reflections
*a* = 13.0977 (9) Å	θ = 1.6–28.0°
*b* = 7.4932 (4) Å	µ = 0.28 mm^−^^1^
*c* = 14.1123 (9) Å	*T* = 296 K
β = 90.149 (5)°	Prism, yellow
*V* = 1385.03 (15) Å^3^	0.80 × 0.38 × 0.08 mm
*Z* = 4	

### Data collection

**Table:**

Stoe IPDS 2 diffractometer	2868 independent reflections
Radiation source: sealed X-ray tube, 12 x 0.4 mm long-fine focus	2267 reflections with *I* > 2σ(*I*)
Plane graphite monochromator	*R*_int_ = 0.028
Detector resolution: 6.67 pixels mm^-1^	θ_max_ = 26.5°, θ_min_ = 1.6°
ω scans	*h* = −16→16
Absorption correction: integration (*X-RED32*; Stoe & Cie, 2002)	*k* = −9→9
*T*_min_ = 0.880, *T*_max_ = 0.978	*l* = −17→17
8760 measured reflections	

### Refinement

**Table:**

Refinement on *F*^2^	Primary atom site location: structure-invariant direct methods
Least-squares matrix: full	Secondary atom site location: difference Fourier map
*R*[*F*^2^ > 2σ(*F*^2^)] = 0.039	Hydrogen site location: inferred from neighbouring sites
*wR*(*F*^2^) = 0.106	H-atom parameters constrained
*S* = 1.04	*w* = 1/[σ^2^(*F*_o_^2^) + (0.0578*P*)^2^ + 0.1445*P*] where *P* = (*F*_o_^2^ + 2*F*_c_^2^)/3
2868 reflections	(Δ/σ)_max_ < 0.001
181 parameters	Δρ_max_ = 0.14 e Å^−^^3^
0 restraints	Δρ_min_ = −0.23 e Å^−^^3^

### Special details

**Table:**

Geometry. Bond distances, angles *etc*. have been calculated using the rounded fractional coordinates. All su's are estimated from the variances of the (full) variance-covariance matrix. The cell e.s.d.'s are taken into account in the estimation of distances, angles and torsion angles
Refinement. Refinement on *F*^2^ for ALL reflections except those flagged by the user for potential systematic errors. Weighted *R*-factors *wR* and all goodnesses of fit *S* are based on *F*^2^, conventional *R*-factors *R* are based on *F*, with *F* set to zero for negative *F*^2^. The observed criterion of *F*^2^ > σ(*F*^2^) is used only for calculating -*R*-factor-obs *etc*. and is not relevant to the choice of reflections for refinement. *R*-factors based on *F*^2^ are statistically about twice as large as those based on *F*, and *R*-factors based on ALL data will be even larger.

### Fractional atomic coordinates and isotropic or equivalent isotropic displacement parameters (Å^2^)

**Table:**

	*x*	*y*	*z*	*U*_iso_*/*U*_eq_	
Cl1	0.16215 (4)	0.21784 (11)	0.00258 (4)	0.0999 (2)	
O1	0.42246 (7)	0.04185 (14)	0.40107 (6)	0.0472 (3)	
O2	0.99398 (9)	0.3320 (2)	0.22281 (11)	0.0764 (5)	
N1	0.58954 (8)	0.10751 (17)	0.41972 (8)	0.0454 (4)	
N2	0.68499 (9)	0.16004 (17)	0.39371 (8)	0.0465 (4)	
N3	0.78867 (9)	0.28188 (18)	0.27895 (9)	0.0499 (4)	
C1	0.42580 (10)	0.17384 (18)	0.20628 (9)	0.0394 (4)	
C2	0.43734 (11)	0.1304 (2)	0.11136 (10)	0.0500 (5)	
C3	0.35657 (13)	0.1423 (2)	0.04893 (11)	0.0585 (5)	
C4	0.26364 (12)	0.1993 (2)	0.08154 (11)	0.0563 (5)	
C5	0.24891 (11)	0.2423 (2)	0.17507 (12)	0.0534 (5)	
C6	0.33006 (10)	0.2283 (2)	0.23759 (10)	0.0455 (4)	
C7	0.51501 (10)	0.16802 (18)	0.27082 (9)	0.0382 (4)	
C8	0.50364 (10)	0.10152 (19)	0.36713 (9)	0.0393 (4)	
C9	0.69357 (10)	0.21801 (19)	0.30668 (10)	0.0423 (4)	
C10	0.60866 (10)	0.22402 (19)	0.24347 (10)	0.0427 (4)	
C11	0.87216 (12)	0.2561 (3)	0.34556 (13)	0.0625 (6)	
C12	0.96240 (13)	0.3681 (3)	0.31683 (15)	0.0724 (7)	
C13	0.91285 (13)	0.3718 (4)	0.16077 (15)	0.0835 (8)	
C14	0.81944 (13)	0.2585 (3)	0.18073 (14)	0.0745 (7)	
H1	0.58330	0.07350	0.47760	0.0550*	
H2	0.50070	0.09260	0.08950	0.0600*	
H3	0.36500	0.11210	−0.01450	0.0700*	
H5	0.18530	0.28040	0.19610	0.0640*	
H6	0.32050	0.25550	0.30120	0.0550*	
H10	0.61770	0.26720	0.18230	0.0510*	
H11A	0.89160	0.13120	0.34680	0.0750*	
H11B	0.85030	0.28970	0.40870	0.0750*	
H12A	0.94450	0.49330	0.32220	0.0870*	
H12B	1.01870	0.34500	0.35990	0.0870*	
H13A	0.93470	0.35190	0.09600	0.1000*	
H13B	0.89500	0.49690	0.16710	0.1000*	
H14A	0.76410	0.29350	0.13890	0.0890*	
H14B	0.83490	0.13390	0.16890	0.0890*	

### Atomic displacement parameters (Å^2^)

**Table:**

	*U*^11^	*U*^22^	*U*^33^	*U*^12^	*U*^13^	*U*^23^
Cl1	0.0672 (3)	0.1570 (6)	0.0753 (3)	−0.0021 (3)	−0.0358 (2)	0.0166 (3)
O1	0.0438 (5)	0.0619 (6)	0.0358 (5)	−0.0068 (5)	0.0003 (4)	0.0016 (4)
O2	0.0386 (5)	0.0910 (10)	0.0996 (10)	0.0011 (6)	0.0060 (6)	0.0097 (8)
N1	0.0441 (6)	0.0552 (7)	0.0370 (6)	−0.0050 (5)	−0.0037 (4)	0.0043 (5)
N2	0.0401 (6)	0.0511 (7)	0.0482 (6)	−0.0027 (5)	−0.0062 (5)	0.0051 (5)
N3	0.0353 (6)	0.0576 (8)	0.0569 (7)	−0.0008 (5)	−0.0028 (5)	0.0103 (6)
C1	0.0399 (7)	0.0394 (7)	0.0390 (6)	−0.0022 (5)	−0.0027 (5)	0.0050 (5)
C2	0.0474 (8)	0.0604 (9)	0.0422 (7)	0.0042 (7)	0.0001 (6)	0.0033 (6)
C3	0.0631 (9)	0.0735 (11)	0.0389 (7)	−0.0033 (8)	−0.0075 (6)	0.0036 (7)
C4	0.0480 (8)	0.0671 (11)	0.0537 (8)	−0.0070 (7)	−0.0159 (6)	0.0131 (7)
C5	0.0378 (7)	0.0626 (10)	0.0598 (9)	0.0002 (6)	−0.0038 (6)	0.0085 (7)
C6	0.0405 (7)	0.0524 (8)	0.0437 (7)	−0.0010 (6)	−0.0017 (5)	0.0017 (6)
C7	0.0389 (6)	0.0372 (7)	0.0386 (7)	0.0030 (5)	−0.0018 (5)	0.0009 (5)
C8	0.0411 (6)	0.0404 (7)	0.0365 (6)	−0.0006 (5)	−0.0019 (5)	−0.0023 (5)
C9	0.0371 (6)	0.0403 (7)	0.0496 (7)	0.0024 (5)	−0.0029 (5)	0.0037 (6)
C10	0.0399 (7)	0.0441 (8)	0.0440 (7)	0.0039 (6)	−0.0010 (5)	0.0079 (6)
C11	0.0414 (8)	0.0720 (11)	0.0740 (11)	−0.0014 (7)	−0.0124 (7)	0.0149 (9)
C12	0.0418 (8)	0.0806 (13)	0.0947 (14)	−0.0067 (8)	−0.0117 (8)	0.0124 (11)
C13	0.0474 (9)	0.1222 (19)	0.0809 (13)	−0.0066 (10)	0.0089 (8)	0.0210 (13)
C14	0.0456 (9)	0.1111 (16)	0.0669 (11)	−0.0097 (9)	0.0074 (8)	0.0022 (11)

### Geometric parameters (Å, º)

**Table:**

Cl1—C4	1.7379 (17)	C7—C8	1.4556 (18)
O1—C8	1.2500 (16)	C7—C10	1.3535 (19)
O2—C12	1.417 (3)	C9—C10	1.4246 (19)
O2—C13	1.407 (2)	C11—C12	1.506 (3)
N1—N2	1.3620 (16)	C13—C14	1.516 (3)
N1—C8	1.3470 (17)	C2—H2	0.9300
N2—C9	1.3079 (18)	C3—H3	0.9300
N3—C9	1.3915 (18)	C5—H5	0.9300
N3—C11	1.453 (2)	C6—H6	0.9300
N3—C14	1.455 (2)	C10—H10	0.9300
N1—H1	0.8600	C11—H11A	0.9700
C1—C2	1.3872 (19)	C11—H11B	0.9700
C1—C6	1.3918 (19)	C12—H12A	0.9700
C1—C7	1.4803 (18)	C12—H12B	0.9700
C2—C3	1.378 (2)	C13—H13A	0.9700
C3—C4	1.371 (2)	C13—H13B	0.9700
C4—C5	1.373 (2)	C14—H14A	0.9700
C5—C6	1.384 (2)	C14—H14B	0.9700
			
C12—O2—C13	108.69 (14)	N3—C14—C13	109.58 (16)
N2—N1—C8	128.84 (11)	C1—C2—H2	119.00
N1—N2—C9	115.48 (11)	C3—C2—H2	119.00
C9—N3—C11	116.43 (13)	C2—C3—H3	120.00
C9—N3—C14	118.48 (13)	C4—C3—H3	120.00
C11—N3—C14	112.98 (13)	C4—C5—H5	120.00
C8—N1—H1	116.00	C6—C5—H5	120.00
N2—N1—H1	116.00	C1—C6—H6	120.00
C2—C1—C7	119.96 (12)	C5—C6—H6	120.00
C2—C1—C6	118.42 (12)	C7—C10—H10	119.00
C6—C1—C7	121.58 (12)	C9—C10—H10	119.00
C1—C2—C3	121.12 (14)	N3—C11—H11A	110.00
C2—C3—C4	119.11 (14)	N3—C11—H11B	110.00
Cl1—C4—C3	119.21 (12)	C12—C11—H11A	110.00
Cl1—C4—C5	119.25 (12)	C12—C11—H11B	110.00
C3—C4—C5	121.54 (15)	H11A—C11—H11B	108.00
C4—C5—C6	119.06 (14)	O2—C12—H12A	109.00
C1—C6—C5	120.73 (13)	O2—C12—H12B	109.00
C8—C7—C10	117.86 (12)	C11—C12—H12A	109.00
C1—C7—C10	122.00 (12)	C11—C12—H12B	109.00
C1—C7—C8	120.14 (11)	H12A—C12—H12B	108.00
O1—C8—N1	120.72 (12)	O2—C13—H13A	109.00
O1—C8—C7	124.71 (12)	O2—C13—H13B	109.00
N1—C8—C7	114.57 (12)	C14—C13—H13A	109.00
N2—C9—N3	117.23 (12)	C14—C13—H13B	109.00
N3—C9—C10	120.73 (13)	H13A—C13—H13B	108.00
N2—C9—C10	121.98 (12)	N3—C14—H14A	110.00
C7—C10—C9	121.21 (13)	N3—C14—H14B	110.00
N3—C11—C12	109.97 (16)	C13—C14—H14A	110.00
O2—C12—C11	112.14 (16)	C13—C14—H14B	110.00
O2—C13—C14	111.97 (19)	H14A—C14—H14B	108.00
			
C13—O2—C12—C11	60.9 (2)	C7—C1—C6—C5	176.41 (13)
C12—O2—C13—C14	−61.1 (2)	C2—C1—C7—C10	39.3 (2)
C8—N1—N2—C9	2.5 (2)	C6—C1—C7—C10	−138.37 (15)
N2—N1—C8—C7	−2.9 (2)	C1—C2—C3—C4	0.5 (2)
N2—N1—C8—O1	176.78 (13)	C2—C3—C4—Cl1	178.98 (12)
N1—N2—C9—C10	−0.5 (2)	C2—C3—C4—C5	−0.9 (2)
N1—N2—C9—N3	176.65 (12)	C3—C4—C5—C6	0.3 (2)
C11—N3—C9—C10	−175.00 (15)	Cl1—C4—C5—C6	−179.64 (12)
C14—N3—C11—C12	51.2 (2)	C4—C5—C6—C1	0.9 (2)
C9—N3—C11—C12	−166.70 (15)	C1—C7—C8—O1	2.1 (2)
C11—N3—C9—N2	7.8 (2)	C10—C7—C8—O1	−178.24 (14)
C14—N3—C9—N2	147.76 (15)	C10—C7—C8—N1	1.46 (19)
C11—N3—C14—C13	−51.3 (2)	C8—C7—C10—C9	0.1 (2)
C14—N3—C9—C10	−35.0 (2)	C1—C7—C10—C9	179.78 (13)
C9—N3—C14—C13	167.46 (16)	C1—C7—C8—N1	−178.23 (12)
C2—C1—C7—C8	−141.00 (14)	N3—C9—C10—C7	−177.67 (13)
C7—C1—C2—C3	−177.11 (13)	N2—C9—C10—C7	−0.6 (2)
C6—C1—C2—C3	0.7 (2)	N3—C11—C12—O2	−55.9 (2)
C6—C1—C7—C8	41.30 (19)	O2—C13—C14—N3	56.5 (2)
C2—C1—C6—C5	−1.3 (2)		

### Hydrogen-bond geometry (Å, º)

**Table:**

*D*—H···*A*	*D*—H	H···*A*	*D*···*A*	*D*—H···*A*
N1—H1···O1^i^	0.86	1.92	2.7705 (15)	170
C5—H5···O2^ii^	0.93	2.56	3.4737 (19)	166
C6—H6···O1	0.93	2.51	2.9538 (17)	109
C10—H10···O1^iii^	0.93	2.43	3.1614 (17)	136
C12—H12*B*···Cl1^iv^	0.97	2.79	3.754 (2)	173

Symmetry codes: (i) −*x*+1, −*y*, −*z*+1; (ii) *x*−1, *y*, *z*; (iii) −*x*+1, *y*+1/2, −*z*+1/2; (iv) *x*+1, −*y*+1/2, *z*+1/2.
